# MicroRNAs and Androgen Receptor: Emerging Players in Breast Cancer

**DOI:** 10.3389/fgene.2019.00203

**Published:** 2019-03-19

**Authors:** Erika Bandini, Francesca Fanini

**Affiliations:** Biosciences Laboratory, Department of Clinical and Experimental Oncology and Hematology, Istituto di Ricovero e Cura a Carattere Scientifico, Istituto Scientifico Romagnolo per lo Studio e la Cura dei Tumori (I.R.S.T.) S.r.l. IRCCS, Meldola, Italy

**Keywords:** microRNAs, androgens, receptor, breast, cancer

## Abstract

Breast cancer (BC) is the most common cause of cancer among women, with a high incidence rate occurrence every year worldwide despite advances in its management. BC is characterized by a spectrum of subtypes which respond differently to treatments due to their biological features, representing the main issue in the control of this type of malignancy. Androgen receptor (AR) is emerging as a target to investigate among hormone receptors, since it seems to play a role at various stages of development of specific BC subsets. For this reason, in recent years AR has become very important in the clinical practice, although its role remains controversial. A number of studies have proposed a correlation between microRNAs (miRNAs), a class of gene expression modulators, and AR in prostate cancer (PC), but there are still few evidences about the relationship between miRNAs and AR in BC. The purpose of this review is to present a state of the art scenario with consideration to the most recent discoveries about miRNAs involved in the AR associated pathogenesis of BC, in order to provide new insights into the role of miRNAs as key drivers in the modulation of AR, and possible actors in the development and progression of BC. Moreover, we consider findings about involvement of AR signaling in all stages of BC, highlighting its association with different subsets of breast carcinomas and with pre- and postmenopausal state of patients.

## Introduction

Breast cancer (BC) is the most common cause of cancer among women and in 2018 in the United States 266,120 new cases and 40,920 deaths have been estimated (Siegel et al., 2018). Despite advances in the management of the disease, a high incidence rate occurs every year worldwide as a consequence of several factors such as socioeconomic differences in the population (Lukong et al., 2017; Dean et al., 2018), ethnicity (DeSantis et al., 2017; Foy et al., 2018), dietary habits (Tabung et al., 2016; Nattenmüller et al., 2018), and disparities in screening programs (Miglioretti et al., 2015). The study of BC has highlighted a substantial tissue heterogeneity, showing several molecular profiles each with distinct clinical and biological features (Perou et al., 2000) which make this tumor differently responsive to treatments, and adverse in its management. In the last years, molecular profiling by gene expression and transcriptional studies has provided an important tool to classify BCs into four well-established subtypes: Luminal A, Luminal B, Basal-like, and human epidermal growth factor receptor 2 (Her2)-enriched (Parker et al., 2009). Among Basal like group, TNBC represents a heterogeneous category of cancer whose immunohistochemical classification lacks of ER, PGR, and HER2 protein expression. Up to day, several studies have been conducted in order to better identify molecular-based therapies. Integrated molecular analyses have significantly enhanced the knowledge about genomic drivers of the most common BC subtypes, giving prominence to the discovery of novel subtype-specific targets that can be exploited in the future especially for the treatment of TNBCs (Koboldt et al., 2012; Burstein et al., 2015). Lehmann et al. analyzed 587 TNBC cases and identified 6 TNBC subtypes displaying unique gene expression profile: two basal-like (BL1 and BL2), an immunomodulatory (IM), a mesenchymal (M), a mesenchymal stem-like (MSL) and a Luminal Androgen Receptor subtype (LAR), composed of AR driven tumors, and that has been suggested to be correlated to the previously identified by Farmer et al. “Molecular Apocrine” subtype (MA) (Farmer et al., 2005; Lehmann et al., 2011; Lehmann-Che et al., 2013). Among these subtypes, LAR type was found to be associated with older patient age, apocrine histologic features, low density of stromal tumor-infiltrating lymphocytes (TIL), and low Ki-67 labeling index (Kim et al., 2018). Sex steroid hormone receptors, ER and PGR, have always played a leading role in the development and progression of BC; however, in the last years, AR has emerged as a prominent player to focus attention on. In terms of therapeutic options, AR may provide a further strategy to counteract breast malignancy, especially in patients with ER negative (ER-) tumors that do not benefit from endocrine or Her2 targeted treatments. In the last decades several small molecules have been identified as critical regulators of transcription and translation of proteins involved in tumorigenesis, and among them miRNAs are the most studied. They belong to a broad family of small non-coding RNAs, and they deserve great attention since they can modify the expression of tumor suppressor genes or oncogenes, affecting signaling pathways of cancer cells. The function of miRNAs in BC has been deeply explored, with the first miRNA signature reported by Iorio et al., and followed by a plethora of studies that have determined a functional role of miRNAs in the disease (Iorio et al., 2005). Anyway, little is known about the emerging dysregulation mechanisms of miRNAs in the context of hormonal signaling, especially for androgens. In this review, we present a state of the art scenario about the role of AR in BC, highlighting the main issues about this “player” that is very debating especially for what concerns its function in different subsets of breast carcinomas. Moreover, we focus on the most recent discoveries about miRNAs involved in the AR associated pathogenesis of BC, since so far this topic has been considered almost exclusively in PC.

## The Role of Androgen Receptor in Breast Cancer

Androgen receptor is a member of the family of steroid nuclear receptors which mediates the biological effects of androgens. It is well established that AR is considered an oncogenic driver at all stages of PC, but its role in BC remains controversial. In fact, expression of AR splice variants (ARVs) has been elucidated in PC, but only recently the presence of multiple known and novel ARV transcripts has been demonstrated in a panel of BC cell lines and human tissues: AR-V1, -V3, -V4, -V7, and -V9 (Hu et al., 2014). In particular, AR-V7 was observed to be constitutively active and involved in androgen deprivation resistance in more than 50% of BC cases (Hickey et al., 2015). Whereas AR and ER-α have a quite similar structure and are co-expressed by many BCs, the role of AR may be different depending on the levels of both hormone receptors in the tumor environment. This becomes important in the evaluation of clinical practice, as in pre-clinical models of BC AR was able either to stimulate or inhibit cell proliferation (Macedo et al., 2006; Lin et al., 2009).

In the last years several studies have focused on the role of AR in ER-α positive (ER+) BCs, since there would seem to be a correlation between its expression grade and some clinical advantages. In fact high levels of AR are associated to reduced lymph node involvement, better DFS, RFS, and OS, response to endocrine therapies and chemotherapy, lower tumor grade, Ki67 expression, smaller tumor size and less necrosis, suggesting for AR a possible role as a tumor-suppressor in malignant breast epithelial cells (Peters et al., 2009; Castellano et al., 2010; Vera-Badillo et al., 2014). Rangel et al. investigated the prognostic impact of AR/ER ratio in 402 ER+ BC patients, showing its inverse relation with aggressiveness of biological features and worse prognosis (Rangel et al., 2018b). Similarly, Basile et al. reported that a high AR/ER ratio seems to be detrimental in BC treated with endocrine therapy (Basile et al., 2017), while in 2 validated BC cohorts, ER+ patients with AR positivity ≥78% had the best survival, and among them those with a ratio of AR: ERα >0.87 exhibited the best outcomes (Ricciardelli et al., 2018). In a study involving 479 BC women, it has been evidenced that in ER+ patients the expression of forkhead box A1 (FOXA1), a pioneer factor which helps the recruitment of ER and AR to their response elements on the genome, was directly correlated to the presence of AR and to better outcome, providing additional knowledge about recurrence (Rangel et al., 2018a). Also Park et al. confirmed that ER+ patients with low expressed AR and FOXA1 tumors were significantly correlated to worse RFS (Park et al., 2017). Moreover, recent data showed that over 90% of metastasis from luminal tumors preserved FOXA1 expression (Ross-Innes et al., 2012), and the concomitant expression of AR and FOXA1 in metastatic lesions may promote the luminal to MA transition. In TNBCs, AR is expressed in 10–43% of cases but its prognostic value remains still unclear. Actually, larger cohort numbers should be needed to determine a role for AR in this peculiar subtype. In some studies involving TNBC cases, the presence of AR appeared correlated to an increase in overall mortality, lymph node metastasis and higher tumor stage (Hu et al., 2011; McGhan et al., 2014). Conversely, another group demonstrated that androgen pathways are associated with reduced aggression TNBC, and that AR loss may have a role in the progression of the tumor (McNamara et al., 2014). A meta-analysis involving 13 studies with 2826 TNBC cases, suggested a potential role of AR in a lower risk of recurrence highlighting that AR positive women showed prolonged DFS (Wang et al., 2016). After analysis of 135 invasive TNBC cases, AR and epidermal growth factor receptor (EGFR) expression was evaluated in order to stratify TNBCs into three risk groups: low risk (AR+ EGFR-) characterized by better prognosis and beneficial from anti-androgen therapies; high risk (AR- EGFR+) with worst prognosis, but better responsiveness to chemotherapy; and intermediate-risk (AR+ EGFR+, AR-, EGFR-) (Astvatsaturyan et al., 2018).

Molecular apocrine subtype has been studied *in vitro* using BC cell lines whose growth was promoted by AR expression. Robinson et al. demonstrated that in the absence of ER-α more than a half of AR binding events showed an analogous pattern to that of ER-α in ER+ cells, promoting the expression of ER target genes, and suggesting a role of AR as a ER-α mimic (Robinson et al., 2011). Anyway, the biological interaction between ER-α and AR still needs to be clarified. Curiously, in a transcriptomic study involving male BC, chromatin binding landscape of *ER* in relation to steroid hormone receptors including *AR*, was determined. Results showed that AR pathway was the only hormonal signaling more associated with the *ER-α* binding genes, confirming that genomic functions of *ER-α* and *AR* in BC are largely overlapping (Severson et al., 2018). For what concerns HER2-enriched BC subtype, it has been found strongly related to MA and studies have suggested a strong evidence of the proliferative role of AR (Ni et al., 2011; Chia et al., 2015). Lehmann-Che et al. tried to characterize MA tumors and found that they were all defined ER-, AR+, FOXA1+, with an overexpression of HER2 or prolactin induced protein (GCDFP15), useful for discriminating MA from basal-like (BL) in the context of ER- tumors. This distinction can be useful to include MA patients in specific “AR pathway” trials, being this subtype rather aggressive (Lehmann-Che et al., 2013). There are evidences that AR can promote *ERK* activation up-regulating *HER2* gene transcription, therefore contributing to the growth of Her2+ BC (Naderi and Hughes-Davies, 2008; Chia et al., 2011). More recently, the functional role of AR was investigated by silencing assays and a reduction in the growth of Her2+ BC cells HCC1954 and SKBr3 was observed, also after treatment with the androgen antagonist Enzalutamide, highlighting a function of AR in promoting the growth of Her2+ BC cells (He et al., 2017). Daemen and Manning explored *HER2* amplification in 3155 breast tumors and found that the HER2–enriched (HER2E) subtype had a distinct transcriptional landscape independent of *HER2*-amplificated (HER2A) that reflected and confirmed how AR signaling can replace ER-driven tumorigenesis (Daemen and Manning, 2018). In a study involving 1297 primary tumors and 336 paired axillary lymph node metastases, Kraby et al. found a highest proportion of AR positivity in the Luminal B subtype while the lowest was observed in the basal phenotype. Interestingly, in 60/72 cases a changeover from AR- primary tumor to AR+ lymph node metastasis occured. Moreover, in primary tumors AR expression was an independent and favorable prognostic marker, particularly in the Luminal A subtype, and in grade 3 tumors (Kraby et al., 2018). All these observations underline the need for a more detailed classification of tumor samples aimed at a more targeted and personalized treatment of patients. The role of AR in BC subtypes is resumed in Table 1.

**Table 1:** The role of AR in BC subtypes.

Tumor subtype	AR role	Reference
**ER+**	Tumor-suppressor: associated with low aggressiveness and better outcome	Peters et al., 2009; Castellano et al., 2010; Vera-Badillo et al., 2014; Basile et al., 2017; Kim et al., 2017; Rangel et al., 2018a,b; Ricciardelli et al., 2018
**TNBC**	Tumor-suppressor: associated with low aggressiveness and progression, and better outcome Oncogenic: associated with aggressiveness and worse outcome	McNamara et al., 2014; [Bibr B81][Bibr B32]; McGhan et al., 2014
**TNBC AR+/EGFR- TNBC AR+/EGFR+ TNBC AR-/EGFR- TNBC AR-/EGFR+**	Associated with better prognosis Group with intermediate risk Associated with worse prognosis	Astvatsaturyan et al., 2018
**MA ER-**	ER mimic	Robinson et al., 2011; Severson et al., 2018
**HER2-enriched**	Proliferative	Naderi and Hughes-Davies, 2008; Ni et al., 2011; Chia et al., 2015; He et al., 2017; Daemen and Manning, 2018
**Luminal A primary tumors**	Favorable prognostic marker	Kraby et al., 2018

AR is expressed in all stages of BC (*in situ*, primary and metastatic). In fact, it is estimated that up to 90% of primary BC and up to 75% of metastatic lesions expressed AR (Hickey et al., 2012), as well as in the 50–80% of invasive BCs and in the 85% of ductal carcinoma *in situ* (DCIS) (Lim et al., 2014), although among the BC subtypes the frequency appears variable. Nevertheless, its role in breast carcinogenesis remains a debated topic as its contribution to the different tumor stages development and progression still needs to be clarified. Feng et al. reported the involvement of DHT in the initiation of epithelial-to-mesenchymal transition (EMT) of BC cells in an AR-dependent but ER-independent manner, indicating the role of androgens in cancer invasion and metastasis (Feng et al., 2017), Schrijver et al. investigated receptor conversion in 91 effusion metastasis, pleural and peritoneal, of 69 patients by immunohistochemistry and *in situ* hybridization. AR receptor status changed from positive in the primary tumor to negative in the effusion metastases or *vice versa* in 46–51% of cases, and this was more often associated in patients previously treated with ET (Schrijver et al., 2017). This new finding could be relevant for investigating AR-targeted therapies in ER- and endocrine resistant BC. RNA sequencing was performed to investigate CTCs isolated from blood samples of patients with metastatic ER+ BC, and a comparison between cases with progression in bone vs. visceral organs was made. Results showed that the most activated pathway in CTCs from bone was that of AR, especially involving splice variant AR-V7. Curiously, AR expression within CTCs was associated with the duration of treatment with aromatase inhibitors (AIs), proposing a possible mechanism in the contribution of acquired resistance to ET, and underlying the role of AR in BC bone metastasis together with the therapeutic option of its targeting in patients with metastatic setting (Aceto et al., 2018). Usually, the maintenance of the balance between DHT, the most potent endogenous AR ligand derived from testosterone (Labrie et al., 2003; Gao et al., 2005), and E2 ensures the physiological response of the breast tissue, including BC tissue, depending on the hormonal needs and the menopausal status. In fact, the circulating androgens concentration varies in woman in relation to pre and postmenopause state (Giovannelli et al., 2018). Whereas after menopause circulating level of E2 decrease dramatically up to 10-fold, androgens begin to acquire an important function (Rothman et al., 2011). Several studies have tried to analyze the correlation between circulating androgens and BC growth since this relationship remains unclear, although up to now a high serum testosterone level has been associated with an increased risk in postmenopausal women. It follows that an additional complication in understanding the role of AR is to be attributed to the menopausal state of patients, which seems to be a more significant variable than age. It would be important to distinguish between the intratumoral estrogen or androgen production, and to take into consideration the balance between these different sex hormones. The most of breast tumors are estrogen-dependent and are characterized by a high expression of ER that could interfere with the activity of AR and *vice versa*. Premenopausal patients BC tissues are characterized by higher production of estrogen, and in these individuals ovary is the main source of E2. Otherwise, in postmenopausal state estrogens derived from circulating adrenal androgens, such as androstenedione, and in these patients BC tissues presents lower levels of E2 and higher androgen levels (Takagi et al., 2018). How hormonal changes influence cancer development is still a discussed issue. Curiously, data showed that in recent decades incidence rates of advanced BC have increased for premenopausal women (Fahlén et al., 2018).

## The Interaction Between miRNAs and Androgen Receptor in Breast Cancer

MicroRNAs are the most explored non-coding RNAs, and give rise to a large family of short (19–24 nucleotides) single-strand RNAs which take part in a variety of biological processes, such as cell proliferation, death, differentiation, and stress response (Bartel, 2004; Kozomara and Griffiths-Jones, 2014). They operate recognizing a 2–7 nucleotides “seed-region” in the target mRNA, which can be localized in the 3′-UTR (Lewis et al., 2005), in the 5′-UTR (Lytle et al., 2007), or in the coding region (Forman et al., 2008). Their regulatory function on gene expression is performed through the control of translation of the mRNA target, which can result in downregulation but also in upregulation of the encoded protein (Ambros, 2004; Vasudevan et al., 2007). A decisive turning point was given by Fabbri et al., who highlighted for the first time the ability of miRNAs secreted by tumor-derived exosomes (TEX) to act as paracrine agonists of a specific receptor family suggesting an involvement in the tumor microenvironment interaction and a new possible target for cancer treatment (Fabbri et al., 2012). On this trail, other groups started to analyze the implication of miRs in tumor communication, growth and spread, and recently it has been demonstrated how breast-cancer TEX are able to carry precursor miRNAs (pre-miRNAs) complexed with Dicer, TRBP and AGO2 proteins displaying a cell-independent capacity to process pre-miRNAs into mature form, contributing to the comprehension of a cell-autonomous process occurring in exosomes when secreted into the extracellular space (Melo et al., 2014).

**Figure 1:**
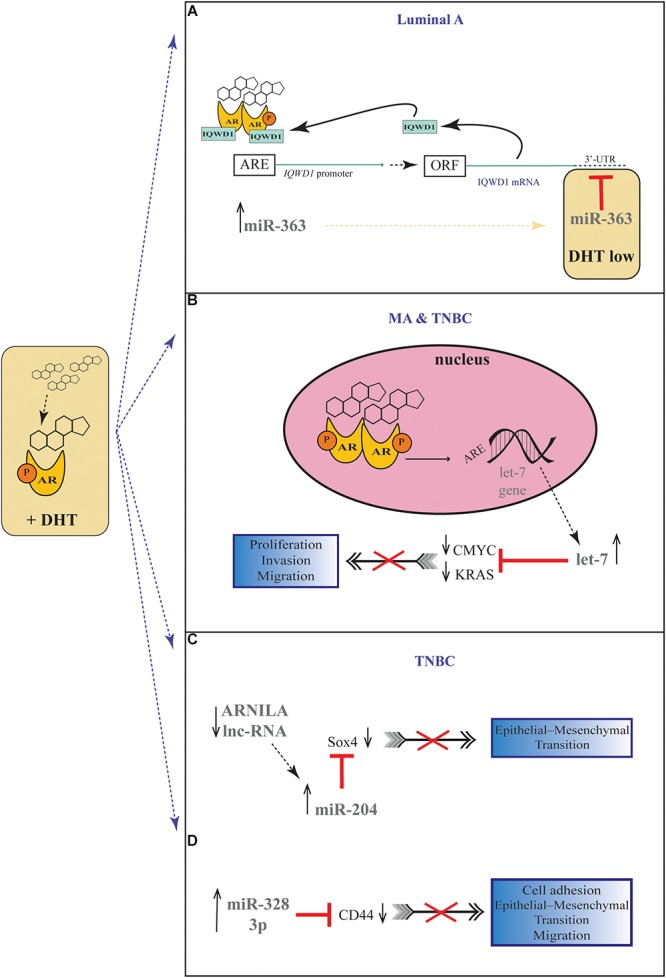
Androgenic-inducible miRNAs involved in the process of BC progression. **(A)** In Luminal A BC cells, DHT treatment induces an androgen-related miRNA-mRNA pathway, involving miR-363 and its possible target gene *IQWD1*. In the presence of high levels of androgens, a IQWD1 feed-forward regulation activates its own AR-mediated expression (Chen et al., 2007), and miR-363 significantly increased. Under relatively low level of DHT, *IQWD1* is negatively regulated by miR-363. **(B)** In MA and TNBC cells, the DHT administration results in an androgen-induced AR activating signal pathway which upregulates let-7 expression and negatively regulates *CMYC* and *KRAS* that are targets of let-7. **(C)** In TNBC cells, the lncRNA ARNILA is negatively regulated by AR after DHT treatment, causing a decreased adsorption of miR-204 which in turn inhibits Sox4 expression, a gene known to promote EMT. **(D)** In TNBC cells, DHT induces upregulation of miR-328-3p with concomitantly decrease of its target CD44, diminishing EMT, migration and adhesion.

**Figure 2:**
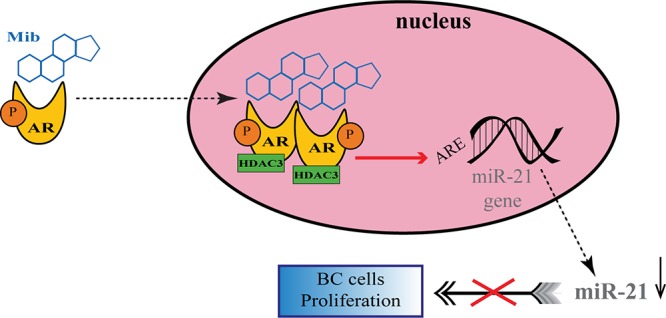
Regulation of onco-miR-21 by AR transcriptional inhibition in BC. Upon miboleron (Mib) binding the AR undergoes a conformational change and translocates to the nucleus where it works as transcriptional inhibitor of onco-miR-21 expression, after recruitment of the co-factor HDAC3. The negative modulation of the onco-mir-21 results in a reduction of BC cells proliferation.

A number of studies have proposed a correlation between miRNAs and AR in PC (Shi et al., 2007; Epis et al., 2009; Ribas et al., 2009; Cao et al., 2010; Nadiminty et al., 2012), but there are still few evidences about the possible role of miRNAs in regulating AR expression in BC. For the first time, Nakano et al., through miRNAs Polymerase Chain reaction (PCR) Arrays, identified miR-363 as an androgen-inducible miRNA. In MCF-7 BC cells they highlighted a possible androgens-related feedback loop involving the gene *IQWD1* (IQ motif and WD repeats-1) and miR-363: under low androgens levels IQWD1 was downregulated by miR-363, but this negative modulation did not occurred after DHT administration (Figure 1A). Interestingly, IQWD1 has a role in protecting AR proteins from degradation *via* proteasome (Nakano et al., 2013). In AR+/ER- models, androgens seemed to mediate a negative correlation between miR-let-7a and the expression of its target oncogenes CMYC and KRAS. In particular, in the MA MDA-MB 453 and in the TNBC MDA-MB 231 cell lines treated with DHT a significant increase in let-7a expression was observed together with a decrease of CMYC and KRAS (Figure 1B). Similarly, in BC tissues the negative correlation was confirmed by IHC, highlighting a new androgen-induced AR activating signal pathway that directly upregulates let-7a and negatively regulates CMYC and KRAS, inhibiting proliferation of AR+/ER- cells (Lyu et al., 2014). Results about the tumor suppressive role of let-7a were confirmed also in AR+/ER+ BC cells, where DHT stimulation led to an AR translocation to the nucleus with transcriptional upregulation of let-7a, decreased cell proliferation, self-renewal capacities, invasion and migration (Zhang et al., 2018). Moreover, in order to deepen the effects of let-7a/AR pathway on breast tumor-initiating cells, Zhang et al. examined the expression of AR, let-7a and CD44^+^/CD24^-/low^ in invasive BC tissues. AR was significantly correlated to let-7a and CD44^+^/CD24^-/low^, highlighting that patients expressing AR and let-7a could have a better outcome, unlike those with a CD44^+^/CD24^-/low^ phenotype which showed a worse prognosis. These findings put in evidence that DHT-induced AR activation plays a critical role in BC, and that AR/let-7a signaling could be exploited as a new optional therapeutic target (Zhang et al., 2018). Another study evidenced the interaction between AR and miRNAs in controlling BC cells behavior. Three BC cell lines (Luminals and MA subtypes) were screened for 84 miRNAs showing each of them a distinct basal miRNAs expression profile. High level of let-7a and -7b found in MA-MDA-453 appeared to be distinctive for MA subtype, whereas miR-205 seemed to represent a marker in the luminal T47D and MCF-7 cells. Furthermore, treatment with the AR agonist CI-4AS-1 led to alterations in the expression profile of other micro-RNAs, such as miR-100 and miR-125 which were found significantly downregulated simultaneously with the increase and extracellular release of metalloprotease-13 (MMP13). Interestingly, the transfection of miR-100 and -125b abrogated the induction of MMP13, suggesting a correlation between these micro-RNAs and AR in the control of BC growth (Ahram et al., 2017). In TNBC, the gene SRY-box 4 (*Sox4*) is known to promote EMT, thereby progression, invasion and metastasis, and is found abnormally overexpressed. Yang et al. identified an AR negatively induced long non-coding RNA (lncRNA) ARNILA that correlated to poor PFS, tightly connected to AR and able to sequester miR-204, in turn facilitating the expression of its target Sox4. Particularly in AR+ carcinomas, ARNILA is suppressed by the action of DHT and AR, resulting in the decreased adsorption of miR-204 thus favoring *Sox4* expression inhibition. On the other hand, in AR- tumors, the action of ARNILA leads to *Sox4* expression by increasing the sequestration of miR-204, leading to the induction of EMT and metastatic propagation (Figure 1C). All together these findings spread light to the discovery of new lncRNA/miRNA/AR mechanisms correlated with poor clinical outcome by regulating EMT, migration, and invasion in TNBC (Yang et al., 2018). Again in TNBC, investigating the modulation of miRNAs after treatment with DHT a group reported miR-328-3p as the most upregulated. Concomitantly, CD44 target of miR-328-3p, decreased, diminishing cell adhesion, migration and EMT, and this result was confirmed also after miR-328-3p mimic transfection (Figure 1D) (Al-Othman et al., 2018). In another study, a total of 153 miRNAs were found to be differentially expressed in AR+ vs. AR- cell lines. The authors identified miR-143, -145, -31, -30c, -30b-3p, 199a, and -181 as significantly downregulated in AR+ cells, while miR-933 and -5793 appeared as the most upregulated, suggesting a role for these miRNAs in the regulation of AR in BC (Shi et al., 2017). Again, the interaction between miR-30a and AR was explored, and miR-30a role was investigated in ER-, PR-, AR+, MDA-MB-453 BC cells. After DHT treatment, which activates Androgen-induced AR signal, a miRNAs profile was identified by miRNAs array, showing a downregulated expression of miR-30a, b and c (among which the most downregulated was miR-30a), and an upregulated expression of AR. Interestingly, in the AR mRNA 3′-untranslated region resides a bioinformatic putative miR-30a, b and c binding site confirming AR as a direct target of miR-30a. Nevertheless, AR does not bind miR-30a promoter region which could be downregulated through other AR-induced cell signaling pathways. This study identified a positive feedback mechanism of regulation which could be explained by two effects. First, the activation of AR expression and AR-induced signal downregulates mir-30a expression that in turn promotes AR availability. Second, the downregulation of mir-30a expression has a negative effect on the inhibition of cell growth induced by itself, being miR-30a a cancer suppressor gene (Lyu et al., 2017). Interestingly, Casaburi et al. reported that androgens can reduce BC cells proliferation by negative modulation of the onco-miR-21. By treatment with synthetic androgen miboleron (Mib) and Chromatin immune-precipitation (ChIP) analysis, they provided evidence that activated AR works as a transcriptional inhibitor of miR-21 expression. In particular, AR was able to bind the proximal promoter of miR-21 in a specific ARE sequence, involving the recruitment of HDAC3 as co-factor in the AR-mediated transcriptional repression (Figure 2). This hypothesis was also supported by a significant reduction of PolII binding in Mib treated extracts, providing further evidence about the protective role of androgens in BC cells (Casaburi et al., 2016).

## Discussion and Future Horizons

Many studies support the idea that BC is a heterogeneous pathology and this consideration is mainly motivated by the existence of different subtypes classified on the basis of hormone receptor expression. About 70–80% of BC express considerable level of ER and are estrogen-dependent (Takagi et al., 2018), but, as well, approximately 70–90% of them express AR, and close to 75% are considered to express both AR and ER (Vera-Badillo et al., 2014) suggesting a role of androgen hormones in the pathogenesis of BC. Although up today the association between levels of circulating androgens and BC risk is still under discussion, several studies identified AR as a marker of favorable prognosis and have demonstrated the anti-proliferative effects of androgens in BC cells (Andò et al., 2002; Lanzino et al., 2010; Takagi et al., 2010). Definitely, in this moment the scientific community can only state that the AR positivity makes more intricate the BC molecular outlook. It has been suggested that circulating androgens may have a role both as independent molecules and as substrate for estrogen synthesis, but limited to AR+/ER+ BC since in AR+/ER- BC they may act in a more homogenous way (Giovannelli et al., 2018). On the basis of different androgens effects on BC, several approaches having AR as a target have been evaluated, including AR agonists and antagonists.

Since the findings that miRNAs play a role in carcinogenesis and are deregulated in several types of tumor, they have obtained a lot of interest about their potential use as therapeutic agents in chemotherapy. Furthermore, the supposing of the existence of a specific uptake can help the purpose to customize miRNAs as therapeutics alone or, more likely, in combination with today’s anti-cancer therapies. Surely more stress should be placed on understanding the balance between AR and ER in relation to the different subtypes, which gives rise to main questions regarding a different response to endocrine therapies in BC. For instance, the interesting positive feedback mechanism identified by Lyu et al. between AR and miR-30a could be a starting point for further studies about the role of miRNAs as a therapeutic predictive markers, besides the identification of other miRs that are able to target AR, or molecules involved in the AR pathway, can certainly help to find more answers about this interaction (Lyu et al., 2017). Also, not to be underestimated is the recent intriguing branch of miRceptor which fits very well in the context of BC as a hormone-dependent tumor, and which is linked to broader themes such as the study of the tumor microenvironment. Consequently, it is reasonable to foresee how the interaction between miRNAs and AR in BC can become in the future an extensively investigated field, in order to increase the treatment chances and try to get much closer to BC personalized therapies.

## Author Contributions

EB wrote the first draft of the manuscript. FF contributed to the writing of the manuscript. FF and EB drew figures and tables. Both authors critically reviewed the manuscript and approved the final version of the manuscript.

## Conflict of Interest Statement

The authors declare that the research was conducted in the absence of any commercial or financial relationships that could be construed as a potential conflict of interest.

## Abbreviations

AR: androgen receptor
BC: breast cancer
DFS: disease-free survival
DHT: dihydrotestosterone
E2: β-estradiol
ER: estrogen receptor
miRNAs: microRNAs
MA: molecular apocrine
OS: overall survival
PC: prostate cancer
PGR: progesterone receptor
RFS: relapse-free survival
TNBC: triple negative breast cancer.
